# Antigenotoxic Effect of *Chamomilla recutita* (L.) Rauschert Essential Oil in Mouse Spermatogonial Cells, and Determination of Its Antioxidant Capacity *in Vitro*

**DOI:** 10.3390/ijms11103793

**Published:** 2010-09-30

**Authors:** Alejandra Hernández-Ceruelos, Eduardo Madrigal-Santillán, José Antonio Morales-González, Germán Chamorro-Cevallos, Martha Cassani-Galindo, Eduardo Madrigal-Bujaidar

**Affiliations:** 1 Laboratorio de Toxicología, Instituto de Ciencias de la Salud, UAEH, Ex-Hacienda de la Concepción. Tilcuautla. Pachuca de Soto, Hgo. Cp 42080, Mexico; E-Mails: ceruelos@hotmail.com (A.H.-C.); eomsmx@yahoo.com.mx (E.M.-S.); jmorales101@yahoo.com.mx (J.A.M.-G); 2 Laboratorio de Genética, Escuela Nacional de Ciencias Biológicas, IPN, Unidad Profesional A. López Mateos, Zacatenco, Av. Wilfrido Massieu. Col Lindavista, D. F. Cp 07738, Mexico; E-Mail: cassani_m@hotmail.com (M.C.-G); 3 Laboratorio de Toxicología Preclínica, Escuela Nacional de Ciencias Biológicas, IPN, Unidad Profesional A. López Mateos, Zacatenco, Av. Wilfrido Massieu, Col Lindavista, D. F. Cp 07738, Mexico; E-Mail: gchamcev@yahoo.com.mx (G.C.-C)

**Keywords:** sister chromatid exchange, DPPH assay, ferric thiocyanate assay, spermatogonia

## Abstract

*Chamomilla recutita* (L.) Rauschert (Asteraceae), popularly known as chamomile, is a plant used in traditional medicine for various therapeutic purposes. Chamomile essential oil (CEO) is particularly known to inhibit the genotoxic damage produced by mutagens in mice somatic cells. The aim of this research was to determine the inhibitory potential of CEO on the genotoxic damage produced by daunorubicin (DAU) in mice germ cells. We evaluated the effect of 5, 50, and 500 mg/kg of essential oil on the rate of sister chromatid exchange (SCE) induced in spermatogonia by 10 mg/kg of the mutagen. We found no genotoxicity of CEO, but detected an inhibition of SCE after the damage induced by DAU; from the lowest to the highest dose of CEO we found an inhibition of 47.5%, 61.9%, and 93.5%, respectively. As a possible mechanism of action, the antioxidant capacity of CEO was determined using the 1,1-diphenyl-2-picrylhydrazyl (DPPH) free radical scavenging method and ferric thiocyanate assays. In the first test we observed a moderate scavenging potential of the oil; nevertheless, the second assay showed an antioxidant capacity similar to that observed with vitamin E. In conclusion, we found that CEO is an efficient chemoprotective agent against the damage induced by DAU in the precursor cells of the germinal line of mice, and that its antioxidant capacity may induce this effect.

## 1. Introduction

Each human cell metabolizes approximately 1012 molecules of oxygen per day to generate ATP; however, about 1% of the process results in the production of reactive oxygen species (ROS). ROS are also generated by exposure to radiation and chemical carcinogens [1]. It is known that ROS can damage nearby cellular components such as DNA, proteins and lipids, and may be an important etiologic factor in degenerative diseases such as cancer, cardiovascular and cerebrovascular pathologies, as well as in the aging process [2]. On the other hand, a number of plant species are currently under research in the chemopreventive field, some of which have revealed valuable results in reducing genetic damage and carcinogenesis; for example the green, black, and white teas have shown positive effects against damage induced by several agents [3,4].

Chamomile (*Chamomilla recutita*) is an asteraceae plant native to Europe and distributed around the world, except in tropical and polar regions. This plant has been used for its curative properties since ancient Egyptian and Greek times, and at present is frequently used as an antiseptic, antiflogistic, diuretic, expectorant, febrifuge, sedative, anti-inflammatory and anticarcinogen [5]. Pharmacological activities of various components of the plant have been reported, for example, the anti-inflammatory capacity and the modulating effects of the heat shock protein on apigenin and quercetin flavonoids, as well as the anti-inflammatory, antioxidant, and antiseptic activities detected on α**-**bisabolol, guargazulene, and chamazulene [6–8].

The essential oil extracted from the chamomile flower varies from 0.42 to 2%, and consists of compounds such as bisabolol, chamazulene, ciclic sesquiterpenes, bisabolol oxides, and other azulenes and terpenes [9,10]. We have demonstrated that chamomile essential oil (CEO) is an effective antigenotoxic agent, producing a dose dependent inhibition of sister chromatid exchanges (SCE) that are induced by daunorubicin (DAU) and methyl methanesulfonate (MMS) in mice bone marrow [11]. In that study, the highest protection of CEO was determined against chromosomal damage induced by DAU, a free radical inducer that is also an intercalating agent and inhibits the topo II enzyme [12]. In order to further determine the antigenotoxic capacity of CEO, in this study, we evaluated its inhibitory potential on the SCE induced by DAU in mouse spermatogonial cells. Moreover, we evaluated the antioxidant capacity of CEO *in vitro* by applying the 1,1-diphenyl-2-picrylhydrazyl (DPPH) free radical scavenging method, and by determining the total antioxidant activity according to the ferric thiocyanate method.

## 2. Material and Methods

### 2.1. Chemicals and Animals

Chamomile essential oil was obtained from Gritman Co. (Frendswood, Tex., U.S.). It was extracted from flowers of *Chamomilla recutita* by steam distillation, and then it was analyzed by gas chromatography to identify the chemical species. Thirteen compounds were determined with this assay, including bisabolol and its oxides, β-farnecene, chamazulene, germacrene, and sesquiterpenes (Table 1).

For the SCE assay, the following chemicals were used: activated charcoal, purchased from Sigma Chemicals (St. Louis, Mo., U.S.), sifted in a sieve (number 200), and neutralized to pH 7.4 with continuous washes of chloridric acid 1M (J.T. Baker, Mexico City), sodium hydroxide 1M (J.T. Baker, Mexico City), and deionized water (Hycell, Mexico City). Corn oil, colchicine, bromodeoxyuridine (BrdU), Hoescht 33258, PBS, EDTA, trypsin, DPPH, α–tocopherol, phosphate buffer, α-cariophylene, and dextrose were purchased from Sigma Chemicals (St. Louis, Mo., U.S.). Linoleic acid was obtained from Fluka Chemicals (St. Louis, Mo., U.S.). Ferrous chloride, iron free ethanol and methanol, amonium thiocyanate, chlorhidric acid, potassium chloride, dibasic sodium phosphate, monobasic potassium phosphate, sodium citrate, citric acid, sodium bicarbonate, sodium chloride, and acetic acid were acquired from J.T. Baker (Mexico City). The Giemsa stain was obtained from Merck (Mexico City), and DAU, 97% pure, from Lemery Laboratories (Mexico City).

The animals used for the SCE study were male mice strain NIH with a mean weight of 25 g. They were obtained from the National Laboratories of Public Health (Mexico City), and maintained in our laboratory in metallic cages at a temperature of 23 °C and 60% humidity, with food (Nutrimix, Mexico City) and water *ad libitum*, in a 12 h light-dark period, adjustment time period for the animals was of one week before the administration of the treatments. The protocol was approved by the Committee of Ethics and Biosecurity of the National School of Biological Sciences.

### 2.2. SCE Assay

Six groups with five animals each were organized for this assay: a negative control group that was administered corn oil orally (0.1 mL), a group positive treated with corn oil (0.1 mL) and DAU administered by intramuscular injection (10 mg/kg), a group administered with 500 mg/kg of CEO, and three groups treated with DAU and CEO (5, 50, and 500 mg/kg) respectively.

Immediately after administration of corn oil and CEO, all animals were treated by intraperitoneal route with an aqueous suspension of BrdU adsorbed to activated charcoal (1.2 mg/kg) so as to obtain the chromatid differential labeling [13]. Twelve hours afterward, 10 mg/kg of DAU was injected to mice of the aforementioned groups. This schedule and route were tested earlier and found to be appropriate to detect the SCE induction by DAU. Colchicine (7.5 mg/kg) was subcutaneously injected 51 h after the beginning of the CEO administration to stop cell division. To obtain spermatogonial cells in metaphase, we followed the technique described by Madrigal-Bujaidar *et al.* [13]. The animals were killed by cervical dislocation and their testes were dissected; the tunica albuginea of each organ was removed, and the seminiferous tubules were fragmented and washed several times with PBS; then, the tissue was put in a 0.1% trypsin solution made in trypsin medium free of calcium and magnesium, plus EDTA; the tissue was agitated for 5 min at 37 °C, dissagregated, centrifuged for 8 min at 2,000 rpm, and the sediment was washed several times with PBS to be finally resuspended in a solution of KCL 0.075 M for 5 min at 37 °C. The supernatant was recovered and centrifuged 5 min at 500 rpm; finally, the cell suspension was fixed three times in a mixture of methanol-acetic acid (3:1) and dropped onto iced slides that were briefly flamed. Differentiation of the sister chromatids was made according to the Hoescht-Giemsa method [13]. Six drops of Hoescht 33250 (2.5 μg/mL) were placed on the slide and expanded with a coverslide and protected from light for 20 min. Buffer solution (phosphate-citrate) was added prior to their exposure to black light for 120 min at a distance of 1 cm. Coverslides were removed by immersion in distilled water and the slides were placed in a Coplin jar with saline citrate solution at 60 °C for 15 min, and washed in hot and cold distilled water prior to staining with a 4% Giemsa solution for 15 min. The microscopic scoring was made in 30 second division metaphases per animal to determine the rate of SCE.

### 2.3. DPPH Radical Scavenging Activity

The method used was the one described by Burtis and Bucar (2000). Methanolic solutions of CEO (1, 5, 10, 100, and 500 mg/mL), α-tocopherol (1 mg/mL), and α-cariophylene (100 mg/mL) were prepared. Then, aliquots of 50 μL of each solution were added to 5 mL of a methanolic solution of DPPH (0.004%) and vortexed. Every assay was repeated 5 times. The absorbance was measured at 517 nm 30 minutes after the addition of the tested substances to the colored reactive.

### 2.4. Ferric Thiocyanate Assay

This assay was made according to the one described by Ono *et al.* (1999). Ethanolic solutions of CEO and α–tocopherol were prepared (14, 140, and 280 mg/mL), then, 200 μL of each solution were added to the reaction mixture: 800 μL of ethanolic solution of linoleic acid 2.25%, 1600 μL of phosphate buffer pH = 7, 600 μL of absolute ethanol (iron free), and 800 μL of deionized water. The mixture was placed in a vial protected from light, and stirred in a vortex. Then, five vials were prepared for each tested concentration of CEO, α-tocopherol, in the linoleic acid control group. The mixtures were incubated at 40 °C for 24, 48, 72, 96, 120, 168, 192, and 226 h, and at each time, 50 μL of each vial was diluted in 4.85 mL of 75% ethanol. Then, we added 50 μL of a 30% ammonium thiocyanate solution, as well as 50 μL of a 20 mM solution of ferrous chloride made in 3.5% chlorhidric acid. The absorbance was measured at 500 nm, 3 min after the addition of ferrous chloride.

### 2.5. Statistical Analysis

Data obtained from the three methods were analyzed using the statistical program INSTAT version 3.0, ANOVA to determine if data were normally distributed within all groups. Thereafter a Student t test was performed to establish the statistical differences between groups in each assay.

## 3. Results

**Figure 1:**
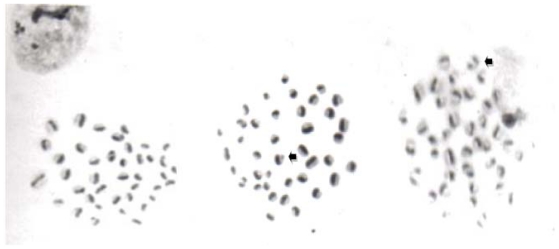
Chromatid differential staining to observe sister chromatid exchange (SCE) in spermatogonial cells. Examples of SCE are indicated by the arrows.

The results obtained in the SCE assay are shown in Figure 1 and Table 2. We found the rate of SCE in mice administered corn oil and CEO in the expected low level of non-mutagenic agents. However, the SCE produced by DAU was more than five-times higher than the values observed for corn oil or CEO. However, animals administered CEO before the DAU treatment showed a significant dose dependent reduction in the SCE rate, for all doses of CEO tested: an inhibition of 47.5%, 61.9%, and 93.5% was determined from the lowest to the highest CEO dose tested.

Table 3 shows the scavenging capacity of CEO determined with the DPPH method. The negative control (α-cariophylene) was unable to decrease the absorbance detected for the DPPH radical, on the contrary, the positive control (1 mg/mL of α-tocopherol) produced an inhibition of 65.81%. With respect to the CEO vials, we found a concentration dependent antioxidant capacity, reaching an inhibition of 80.35% with the highest concentration (500 mg/mL).

**Figure 2:**
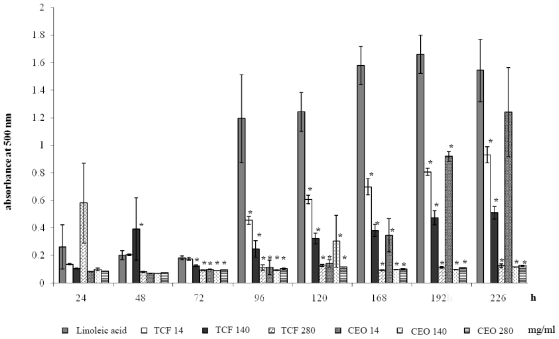
Total antioxidant activity of α-tocopherol and chamomile essential oil (CEO) evaluated with the ferric thiocyanate assay. TCF = α-tocopherol. * Statistically significant difference with respect to the value of the linoleic acid group. ANOVA and Student t tests, *p* < 0.05.

Figure 2 represents the kinetics of oxidation of linoleic acid, and the inhibition of this process by α-tocopherol and CEO. It shows that the inhibition of autooxidation of linoleic acid was significantly decreased by the addition of both chemicals (CEO, and α-tocopherol) in a concentration dependent manner from 72 to 192 h. No significant differences between the results obtained from these two agents were observed. The maximum inhibition percentage (92.1% and 91%) was observed at 96 h with 140 mg/mL and 280 mg/mL of CEO, respectively.

## 4. Discussion

The majority of chemopreventive studies using plant extracts have been made in somatic cells. However, the inhibition of germ cell alterations is also important because of the potential relationship with gonadal tumors and reproductive damage. Anthracyclins such as adriamycin, a chemical structurally related to DAU, and etoposide, are known to alter male germ cells [16]; moreover, rat A1 spermatogonia have been reported to be highly sensitive to adriamycin [17]. Besides, this chemical increases apoptosis in spermatogonia, as well as in preleptotene, zygotene, and pachytene spermatocytes [18]. In our assay for measuring the induction of SCE in spermatogonial cells, DAU showed a strong effect, similar to that previously reported in bone marrow cells [11]. Thus, the chemical was confirmed as a potent SCE inducer in somatic and germ cells. Similarly, in the present study, as well as in the report mentioned above, CEO showed SCE levels in the range of the negative controls. These results establish the absence of a potential genotoxicity *in vivo*, which concurs with the absence of any acute toxicity and collateral damage in consumers of chamomile.

Essential oils of plants are mixtures formed by a variety of chemicals, including terpenes, compounds that are related to different biological effects, such as anti-inflammatory, antioxidant and antimutagenic activities [8,19,20]. In the mixture studied, terpenes, such as bisabolol and its oxides, as well as chamazulene, could be involved in the inhibition of chromosome damage that was observed. In this respect, it is interesting to note that even the low CEO dose tested reduced the SCE formed by DAU, particularly because this amount of oil is consumed in 10 to 12 cups of chamomile tea, a quantity similar to the suggested daily consumption of green tea for reducing the incidence of cancer [21]. With regard to the protection produced by CEO, the effect could have been aided by an easy crossing of the mixture through the testicular barrier resulting from the liposoluble components of the oil [22].

The antioxidant capacity of CEO was evaluated using two *in vitro* methods in an attempt to explore if this effect is relevant for the chemoprotective action of the oil. The DPPH assay is designed to determine the free radical scavenging capacity of a substance, and DAU can act as an inducer of free radicals increasing the oxidative damage to DNA through various pathways [12], in particular through the formation of a semiquinone free radical that under toxic conditions forms superoxide radicals in a redoxcycling reaction [23]. The tested concentrations of CEO showed a positive but moderate effect in this test. It was necessary to use higher concentrations of the oil to reach an inhibition similar to the observed in α-tocopherol. Chamazulene may have influenced the CEO effects as it is one of the major components of the oil with a reductor potential in an iron free system, although the chemical has a lower scavenging capacity than its action over peroxil and hydroxil radicals [19]. Our results agree with previous reports on the determination of the antioxidant activity of commercial essential oils of chamomile, when tested with the agar plate-base method (which includes α-carotene and linoleic acid). The antioxidant values, reported as the retained color and diameter of color retention zone, showed a modest activity for the German chamomile oils [24].

On the other hand, the ferric thiocyanate assay determines the total antioxidant activity of a substance measuring the inhibition of the amount of peroxide produced during the initial stages of oxidation. The high absorbance indicates the autooxidation of the linoleic acid emulsion due to the formation of alcoxy and hydrogen peroxide radicals [25,26]. In our assay, the total antioxidant capacity of CEO was similar to that observed with α-tocopherol at the same concentrations. Chamomile oil seems to inhibit the linoleic acid oxidation during the initial steps of the process, and its effectiveness may be due to a synergistic action of its components. Besides functioning as scavengers, antioxidants are also known to work in the manner mentioned above, thereby producing a broad spectrum of antioxidative activities that create an effective defense system against free radical attack [27,28].

## 5. Conclusion

In the present investigation we demonstrated that treatment with CEO significantly prevents the damage induced by DAU in mice, thereby reducing the level of spermatogonial SCE. Our data also suggest that the antioxidant capacity of the oil may be involved in that effect.

## Figures and Tables

**Table 1:** Components of the tested chamomile essential oil [11].

Compound	RT^a^	CAS no.	Area (%)
(*E*)-β-Farnecene	38.46	28973-97-9	28.17
Germacrene-d	39.23	23986-74-5	2.19
Unidentified sesquiterpene	40.07		1.40
Unidentified sesquiterpene	41.17		0.78
(*Z,E*)-α-Farnecene	41.35	26560-14-5	1.59
Unidentified sesquiterpene	48.52		0.71
α-Bisabolol oxide A	54.46	22567-36-8	41.77
α-Bisabolol oxide B	49.28	26184-88-3	4.31
α-Bisabolol oxide	50.65	22567-38-0	5.30
α-Bisabolol	51.18	515-69-5	2.31
Chamazulene	52.80	529-05-5	2.39
1,6-Dioxaspiro[4,4]non-3-ene,2-(2,4hexadyn-1-ylidene)	60.73		2.19
Hexatriacontane	67.49	630-06-8	0.50

a RT, Retention time obtained with gas chromatography.

**Table 2:** Sister chromatid exchanges (SCE) in spermatogonial cells of mice treated with chamomile essential oil (CEO) and daunorrubicin (DAU).

Agent	Dose (mg/kg)	SCE ± S.D.	Inhibition (%)
Control^a^	0	* 1.90 ± 0.40	
CEO	500	* 2.36 ± 0.44	
DAU	10	11.21 ± 1.38	
CEO+DAU	5+10	* 6.78 ± 1.05	47.5
CEO+DAU	50+10	* 5.44 ± 0.33	61.9
CEO+DAU	500+10	* 2.50 ± 0.31	93.5

a Corn oil (0.1 mL/mouse);

* Statistically significant difference with respect to the group treated with DAU. ANOVA and Student t tests, *p* < 0.05. % inhibition = 100 − (SCE of CEO + DAU - SCE control/SCE of DAU-SCE control) × 100.

**Table 3:** Free radical scavenging capacity of chamomile essential oil (CEO) determined with the DPPH assay.

Agent	Concentration(mg/mL)	Absorbance OD (517 nm)	Absorbance Inhibition (%)
DPPH	0.04	1.059 ± 0.001	
α-cariophylene	100	1.048 ± 0.009	1.0
α-tocopherol	1	0.362 ± 0.015*	65.8
CEO	5	1.057 ± 0.003	0.001
CEO	10	1.013 ± 0.004*	4.3
CEO	50	0.872 ± 0.014*	17.6
CEO	100	0.621 ± 0.022*	41.3
CEO	500	0.208 ± 0.013*	80.4

* Statistically significant difference with respect to the value obtained with DPPH. ANOVA and Student t tests, *p* < 0.05. % inhibition = 1 − (OD value of experimental group/OD value of DPPH group) × 100.
